# Crystal structure of *N*-(2,2,2-tri­chloro-1-hy­droxy­eth­yl)formamide

**DOI:** 10.1107/S2056989015020459

**Published:** 2015-11-14

**Authors:** Mahimaidoss Baby Mariyatra, Helen Stoeckli-Evans

**Affiliations:** a Department of Chemistry, St. Xavier’s College, Palayamkottai 627 002, India; b Institute of Physics, University of Neuchâtel, rue Emile-Argand 11, CH-2000 Neuchâtel, Switzerland

**Keywords:** crystal structure, tri­chloro­hydroxy­eth­yl, formamide, hydrogen bonding

## Abstract

The title compound, C_3_H_4_Cl_3_NO_2_, crystallized with two independent mol­ecules (*A* and *B*) in the asymmetric unit. The two mol­ecules have the same conformation; the mol­ecular overlap gives weighted and unit-weight r.m.s. fits of 0.047 and 0.043 Å, respectively. The conformation of the *N*-(hydroxeth­yl)formamide chains are very similar, as indicated by the C—N(H)—C=O and C—N(H)—C—O(H) torsion angles, which are, respectively, −1.8 (3) and −91.5 (2)° for mol­ecule *A*, and −2.1 (3) and −95.7 (2)° for mol­ecule *B*. In the crystal, individual mol­ecules are linked by pairs of O—H⋯O hydrogen bonds, forming *A*–*A* and *B*–*B* inversion dimers with *R*_2_^2^(12) ring motifs. The dimers are linked *via* N—H⋯O hydrogen bonds, forming alternating layers of *A* and *B* mol­ecules parallel to the *bc* plane. Within the layers of *B* mol­ecules, there are weak C—H⋯Cl hydrogen bonds present.

## Abstract 2

The title compound, crystallized with two conformationally similar mol­ecules (*A* and *B*) in the asymmetric unit. In the crystal, individual mol­ecules are linked by pairs of O—H⋯O hydrogen bonds, forming *A*–*A* and *B*–*B* inversion dimers with 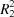
(12) ring motifs.

## Chemical context   

The skeletal structure of formamide is present in a number of medicinally important compounds. This has led to the use of formamides as key inter­mediates in numerous organic synthetic endeavours (Kobayashi *et al.*, 1995 ▸; Chen *et al.*, 2000 ▸; Jackson & Meth-Cohn, 1995 ▸). While formamides are useful formyl­ating agents they have also found utility as easily accessible Lewis bases for promoting several organic transformations (Kobayashi & Nishio, 1994 ▸). Furthermore, in peptide synthesis the formyl group is a valued amino-protecting group (Martinez & Laur, 1982 ▸; Kraus, 1973 ▸). The title compound and related mol­ecules have been found mentioned in several old patent literatures owing to their biocidal properties; both herbicidal (Schiewald *et al.*, 1974 ▸) and fungicidal (Summers & Carter, 1977 ▸) action is known. The title compound is easily obtained by the reaction of 2,2,2-tri­chloro­acetaldehyde and formamide (Sethi, 2006 ▸) and we describe herein its crystal structure.
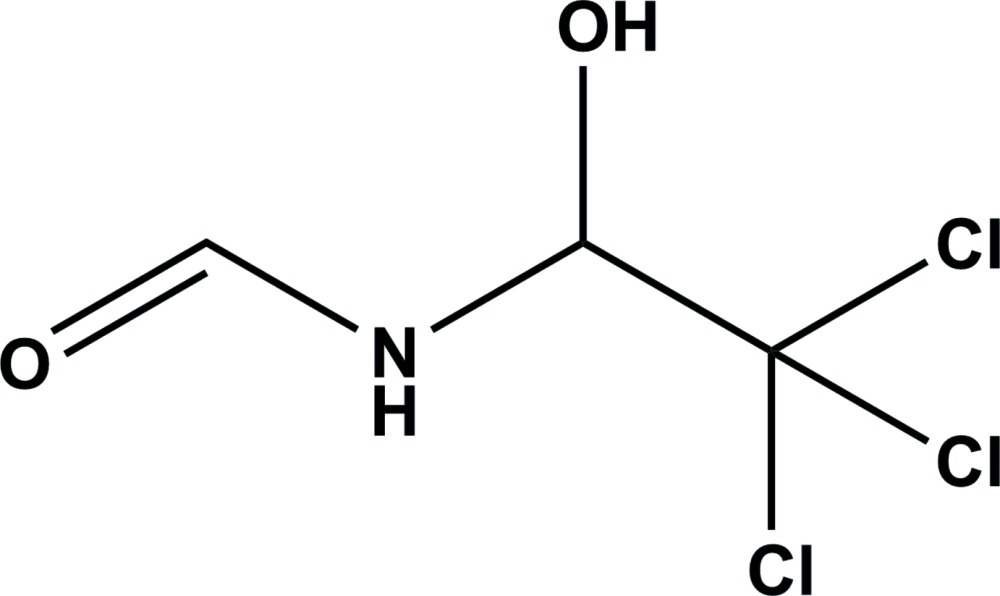


## Structural commentary   

**Figure 1:**
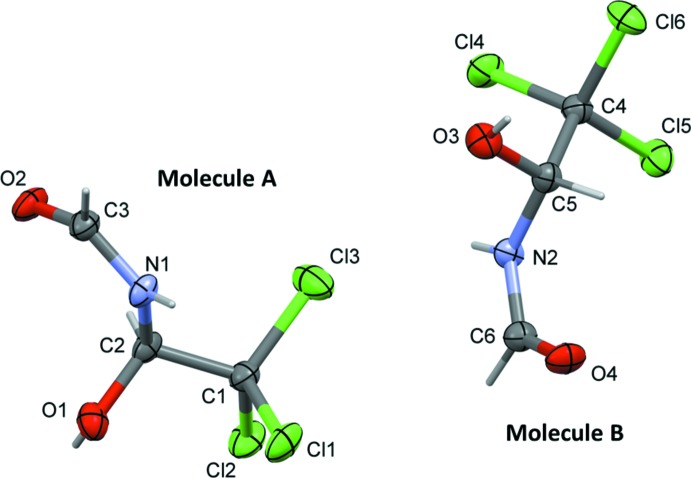
The mol­ecular structure of the two independent mol­ecules (*A* and *B*) of the title compound, showing the atom labelling. Displacement ellipsoids are drawn at the 50% probability level. The torsion angles C2—N1—C3—O2 and C3—N1—C2—O1 are −1.8 (3) and −91.5 (2)°, respectively, for mol­ecule *A*, and C5—N2—C6—O4 and C6—N2—C5—O3 are −2.1 (3) and −95.7 (2)°, respectively, for mol­ecule *B*.

**Figure 2:**
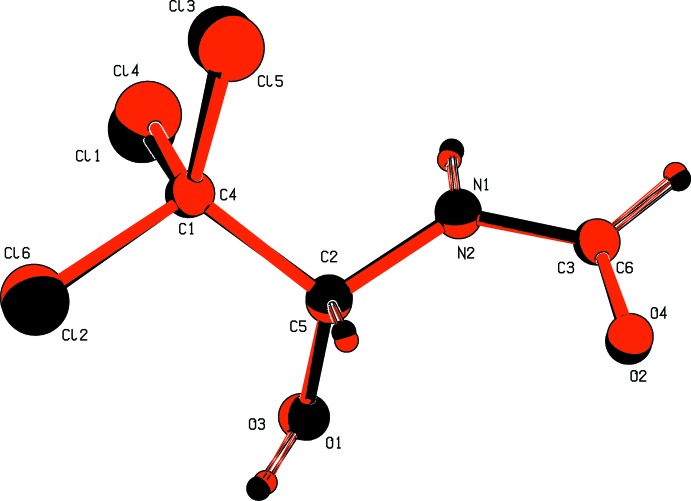
A view of the mol­ecular fit of the six backbone atoms (O1/O3, C1/C4, C2/C5, N1/N2, C3/C6 and O2/O4) of the *A* (black) and *B* (red) mol­ecules of the title compound, calculated using the MolFit routine in *PLATON* (Spek, 2009 ▸).

The title compound, Fig. 1 ▸, crystallized with two independent mol­ecules (*A* and *B*) in the asymmetric unit. The arbitrarily chosen chirality of atoms C2 in mol­ecule *A* and C5 in mol­ecule *B* is the same. The backbones of the two mol­ecules (O1/O3, C1/C4, C2/C5, N1/N2, C3/C6 and O2/O4) have almost identical conformations with weighted and unit-weight r.m.s. overlay fits of 0.047 and 0.043 Å, respectively, for the six atoms in each mol­ecule (Fig. 2 ▸).

## Supra­molecular features   

**Figure 3:**
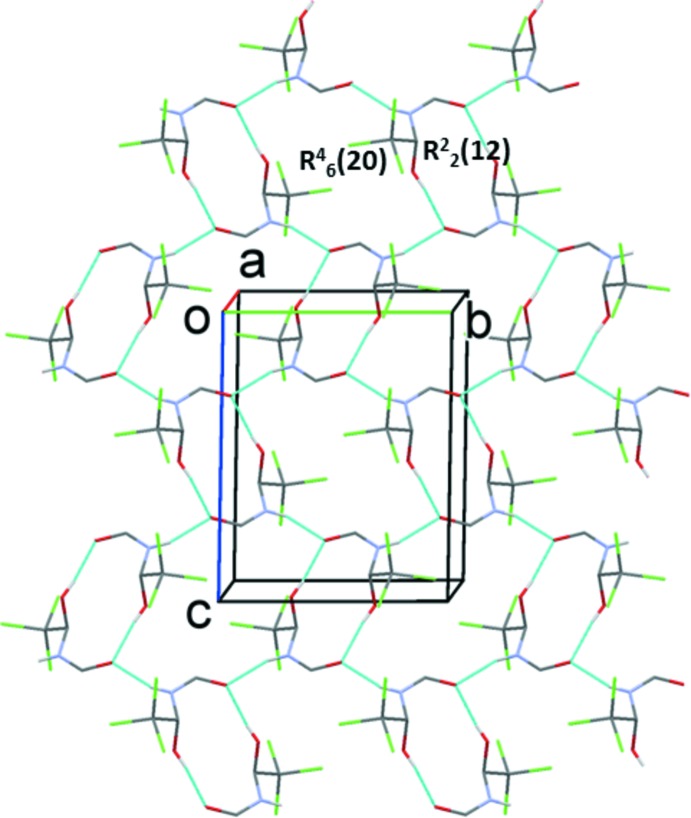
A view along the *a* axis of the hydrogen-bonded layer of *A* mol­ecules of the title compound. Hydrogen bonds are shown as dashed lines (see Table 1 ▸) and C-bound H atoms have been omitted for clarity.

**Figure 5:**
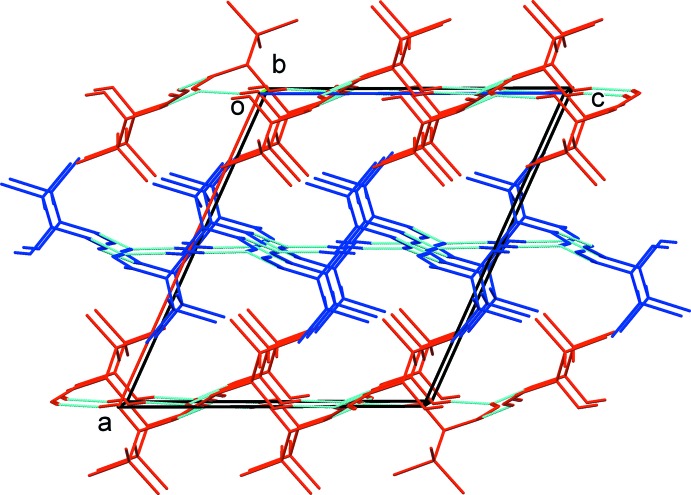
A view along the *b* axis of the crystal packing of the title compound, showing the alternating layers of hydrogen-bonded *A* (blue) and *B* (red) mol­ecules. Hydrogen bonds are shown as dashed lines (see Table 1 ▸) and C-bound H atoms have been omitted for clarity.

In the crystal, the individual mol­ecules are linked by pairs of O—H⋯O hydrogen bonds, forming *A*–*A* and *B*–*B* inversion dimers with 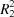
(12) ring motifs (Table 1 ▸ and Figs. 3 ▸ and 4 ▸). The dimers are linked *via* N—H⋯O hydrogen bonds, forming layers of *A* and *B* mol­ecules parallel to the *bc* plane (Table 1 ▸ and Figs. 3 ▸ and 4 ▸). These latter hydrogen bonds lead to the formation of 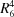
(20) ring motifs in each layer (Figs. 3 ▸ and 4 ▸). The layers stack alternately along the *a* axis, as shown in Fig. 5 ▸. Within the layers of *B* mol­ecules there are weak C—H⋯Cl hydrogen bonds present (Table 1 ▸). There are no significant inter­molecular inter­actions linking the layers.

## Database survey   

A search of the Cambridge Structural Database (CSD, Version 5.36, last update May 2015; Groom & Allen, 2014 ▸) for the acyclic substructure C(=O)—N(H)—C(OH), *viz. N*-(hydroxmeth­yl)formamide, yielded 25 hits. The majority concern metal complexes of the ligand *N*-(hy­droxy­meth­yl)nicotinamide. Only one compound, *N*,*N*′-(1,2-di­hydroxy­ethyl­ene)diformamide (OGEJUG; Taheri & Moosavi, 2008 ▸) resembles the title compound. In the solid state, the whole molecule of this compound is generated by inversion symmetry. The geometric parameters are similar to those observed for the title compound, for example the conformation of the *N*-(hydroxmeth­yl)formamide chain as indicated by the C—N(H)—C—O(H) and C—N(H)—C=O torsion angles: 1.6 (2) and −99.09 (14)° for the above mentioned compound compared to −1.8 (3) and −91.5 (2)° for mol­ecule *A* and −2.1 (3) and −95.7 (2)° for mol­ecule *B* of the title compound (see Fig. 1 ▸).

## Synthesis and crystallization   

The title compound can be synthesized following a literature procedure (Sethi, 2006 ▸), by the reaction of 2,2,2-tri­chloro­acetaldehyde and formamide. An old and discoloured sample of *N-*(2,2,2-tri­chloro-1-hy­droxy­eth­yl)formamide was dissolved in hot ethanol, followed by treatment with charcoal. The filtered solution was left to crystallize by slow evaporation, forming colourless block-like crystals (m.p. 393 K).

## Refinement   

Crystal data, data collection and structure refinement details are summarized in Table 2 ▸. All of the H atoms were located from difference Fourier maps and freely refined.

## Supplementary Material

Crystal structure: contains datablock(s) I. DOI: 10.1107/S2056989015020459/hg5462sup1.cif

Structure factors: contains datablock(s) I. DOI: 10.1107/S2056989015020459/hg5462Isup2.hkl

Supporting information file. DOI: 10.1107/S2056989015020459/hg5462Isup3.cml

CCDC reference: 1009715

Additional supporting information:  crystallographic information; 3D view; checkCIF report

## Figures and Tables

**Figure 4:**
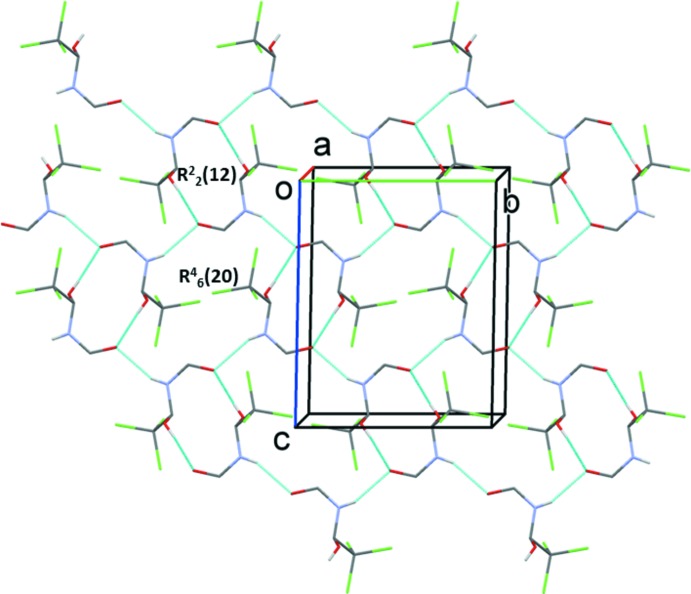
A view along the *a* axis of the hydrogen-bonded layer of *B* mol­ecules of the title compound. Hydrogen bonds are shown as dashed lines (see Table 1 ▸) and C-bound H atoms have been omitted for clarity.

**Table 1:** Hydrogen-bond geometry (Å, °)

*D*—H⋯*A*	*D*—H	H⋯*A*	*D*⋯*A*	*D*—H⋯*A*
O1—H1*O*⋯O2^i^	0.84 (3)	1.90 (4)	2.731 (2)	169 (3)
N1—H1*N*⋯O2^ii^	0.85 (2)	2.08 (3)	2.893 (2)	159 (2)
O3—H3*O*⋯O4^iii^	0.76 (3)	1.97 (3)	2.721 (2)	174 (3)
N2—H2*N*⋯O4^iv^	0.78 (3)	2.17 (3)	2.917 (2)	158 (2)
C6—H6⋯Cl4^v^	1.00 (2)	2.91 (2)	3.586 (2)	125 (2)

Symmetry codes: (i) 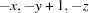; (ii) 
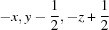
; (iii) 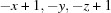; (iv) 
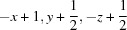
; (v) 
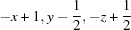
.

**Table 2:** Experimental details

Crystal data	
Chemical formula	C_3_H_4_Cl_3_NO_2_
*M*_r_	192.42
Crystal system, space group	Monoclinic, *P*2_1_/*c*
Temperature (K)	173
*a*, *b*, *c* (Å)	13.7964 (8), 9.0798 (7), 12.2453 (7)
β (°)	114.413 (4)
*V* (Å^3^)	1396.80 (16)
*Z*	8
Radiation type	Mo *K*α
μ (mm^−1^)	1.24
Crystal size (mm)	0.45 × 0.43 × 0.40

Data collection	
Diffractometer	Stoe IPDS 2
Absorption correction	Multi-scan (*MULABS* in *PLATON*; Spek, 2009 ▸)
*T*_min_, *T*_max_	0.579, 1.000
No. of measured, independent and observed [*I* > 2σ(*I*)] reflections	16347, 2645, 2468
*R*_int_	0.056
(sin θ/λ)_max_ (Å^−1^)	0.610

Refinement	
*R*[*F* ^2^ > 2σ(*F* ^2^)], *wR*(*F* ^2^), *S*	0.030, 0.078, 1.08
No. of reflections	2645
No. of parameters	196
H-atom treatment	All H-atom parameters refined
Δρ_max_, Δρ_min_ (e Å^−3^)	0.82, −0.55

Computer programs: *X-AREA* and *X-RED32* (Stoe & Cie, 2009 ▸), *SHELXS2014* (Sheldrick, 2008 ▸), *SHELXL2014* (Sheldrick, 2015 ▸), *PLATON* (Spek, 2009 ▸) and *Mercury* (Macrae *et al.*, 2008 ▸).

## supplementary crystallographic information

### Crystal data

**Table:**

C_3_H_4_Cl_3_NO_2_	*F*(000) = 768
*M**_r_* = 192.42	*D*_x_ = 1.830 Mg m^−^^3^
Monoclinic, *P*2_1_/*c*	Mo *K*α radiation, λ = 0.71073 Å
*a* = 13.7964 (8) Å	Cell parameters from 22309 reflections
*b* = 9.0798 (7) Å	θ = 1.6–26.2°
*c* = 12.2453 (7) Å	µ = 1.24 mm^−^^1^
β = 114.413 (4)°	*T* = 173 K
*V* = 1396.80 (16) Å^3^	Block, colourless
*Z* = 8	0.45 × 0.43 × 0.40 mm

### Data collection

**Table:**

Stoe IPDS 2 diffractometer	2645 independent reflections
Radiation source: fine-focus sealed tube	2468 reflections with *I* > 2σ(*I*)
Plane graphite monochromator	*R*_int_ = 0.056
φ + ω scans	θ_max_ = 25.7°, θ_min_ = 1.6°
Absorption correction: multi-scan (*MULABS* in *PLATON*; Spek, 2009)	*h* = −15→16
*T*_min_ = 0.579, *T*_max_ = 1.000	*k* = −11→11
16347 measured reflections	*l* = −14→14

### Refinement

**Table:**

Refinement on *F*^2^	Hydrogen site location: difference Fourier map
Least-squares matrix: full	All H-atom parameters refined
*R*[*F*^2^ > 2σ(*F*^2^)] = 0.030	*w* = 1/[σ^2^(*F*_o_^2^) + (0.0333*P*)^2^ + 1.2964*P*] where *P* = (*F*_o_^2^ + 2*F*_c_^2^)/3
*wR*(*F*^2^) = 0.078	(Δ/σ)_max_ < 0.001
*S* = 1.08	Δρ_max_ = 0.82 e Å^−^^3^
2645 reflections	Δρ_min_ = −0.55 e Å^−^^3^
196 parameters	Extinction correction: *SHELXL2014* (Sheldrick, 2015), Fc^*^=kFc[1+0.001xFc^2^λ^3^/sin(2θ)]^-1/4^
0 restraints	Extinction coefficient: 0.0089 (9)

### Special details

**Table:**

Geometry. All e.s.d.'s (except the e.s.d. in the dihedral angle between two l.s. planes) are estimated using the full covariance matrix. The cell e.s.d.'s are taken into account individually in the estimation of e.s.d.'s in distances, angles and torsion angles; correlations between e.s.d.'s in cell parameters are only used when they are defined by crystal symmetry. An approximate (isotropic) treatment of cell e.s.d.'s is used for estimating e.s.d.'s involving l.s. planes.

### Fractional atomic coordinates and isotropic or equivalent isotropic displacement parameters (Å^2^)

**Table:**

	*x*	*y*	*z*	*U*_iso_*/*U*_eq_	
Cl1	0.13449 (5)	0.04880 (6)	0.08600 (5)	0.03428 (17)	
Cl2	0.22308 (4)	0.28605 (6)	−0.00018 (5)	0.03123 (16)	
Cl3	0.28102 (5)	0.25302 (8)	0.25345 (5)	0.04298 (19)	
O1	−0.00275 (12)	0.31695 (18)	−0.01499 (14)	0.0284 (4)	
H1O	0.005 (3)	0.366 (4)	−0.069 (3)	0.058 (10)*	
O2	0.00144 (13)	0.53996 (16)	0.21058 (14)	0.0295 (4)	
N1	0.04737 (14)	0.30393 (19)	0.18967 (16)	0.0213 (4)	
H1N	0.0392 (18)	0.215 (3)	0.207 (2)	0.020 (6)*	
C1	0.17653 (17)	0.2341 (2)	0.10963 (19)	0.0234 (4)	
C2	0.08245 (16)	0.3364 (2)	0.09670 (18)	0.0217 (4)	
H2	0.1107 (18)	0.434 (3)	0.1068 (19)	0.020 (6)*	
C3	0.00885 (17)	0.4079 (2)	0.23751 (18)	0.0237 (4)	
H3	−0.0156 (18)	0.374 (3)	0.299 (2)	0.026 (6)*	
Cl4	0.64204 (5)	0.45558 (5)	0.52480 (5)	0.03177 (16)	
Cl5	0.78172 (4)	0.22393 (6)	0.51229 (5)	0.02994 (15)	
Cl6	0.71719 (4)	0.23240 (7)	0.70803 (5)	0.03280 (16)	
O3	0.49315 (12)	0.20023 (18)	0.51142 (15)	0.0274 (3)	
H3O	0.497 (2)	0.153 (3)	0.564 (3)	0.036 (8)*	
O4	0.50422 (13)	−0.04575 (15)	0.29695 (13)	0.0281 (3)	
N2	0.54593 (14)	0.19299 (19)	0.35510 (15)	0.0208 (4)	
H2N	0.5415 (19)	0.274 (3)	0.332 (2)	0.023 (6)*	
C4	0.67565 (16)	0.2671 (2)	0.55274 (18)	0.0216 (4)	
C5	0.57819 (16)	0.1686 (2)	0.48142 (18)	0.0206 (4)	
H5	0.6025 (16)	0.066 (2)	0.5015 (18)	0.012 (5)*	
C6	0.51071 (16)	0.0854 (2)	0.27421 (18)	0.0230 (4)	
H6	0.4911 (19)	0.119 (3)	0.190 (2)	0.028 (6)*	

### Atomic displacement parameters (Å^2^)

**Table:**

	*U*^11^	*U*^22^	*U*^33^	*U*^12^	*U*^13^	*U*^23^
Cl1	0.0493 (4)	0.0157 (3)	0.0449 (3)	0.0062 (2)	0.0265 (3)	0.0024 (2)
Cl2	0.0363 (3)	0.0317 (3)	0.0356 (3)	0.0029 (2)	0.0248 (2)	0.0016 (2)
Cl3	0.0250 (3)	0.0729 (5)	0.0282 (3)	0.0012 (3)	0.0081 (2)	−0.0095 (3)
O1	0.0292 (8)	0.0315 (9)	0.0259 (8)	0.0064 (6)	0.0127 (7)	0.0100 (7)
O2	0.0478 (10)	0.0170 (7)	0.0307 (8)	0.0078 (6)	0.0232 (7)	0.0031 (6)
N1	0.0268 (9)	0.0138 (8)	0.0279 (9)	0.0012 (7)	0.0159 (7)	0.0025 (7)
C1	0.0259 (10)	0.0227 (10)	0.0236 (10)	0.0014 (8)	0.0121 (9)	−0.0014 (8)
C2	0.0272 (10)	0.0144 (10)	0.0275 (10)	0.0016 (8)	0.0154 (8)	0.0010 (8)
C3	0.0288 (11)	0.0221 (10)	0.0229 (10)	0.0034 (8)	0.0133 (9)	0.0013 (8)
Cl4	0.0415 (3)	0.0152 (2)	0.0346 (3)	−0.0028 (2)	0.0117 (2)	−0.0021 (2)
Cl5	0.0247 (3)	0.0366 (3)	0.0304 (3)	0.0009 (2)	0.0133 (2)	0.0020 (2)
Cl6	0.0336 (3)	0.0425 (3)	0.0191 (3)	−0.0029 (2)	0.0077 (2)	0.0028 (2)
O3	0.0287 (8)	0.0303 (8)	0.0265 (8)	−0.0020 (6)	0.0150 (7)	0.0051 (7)
O4	0.0426 (9)	0.0170 (7)	0.0273 (8)	−0.0055 (6)	0.0171 (7)	−0.0019 (6)
N2	0.0271 (9)	0.0132 (8)	0.0202 (9)	−0.0017 (7)	0.0080 (7)	0.0025 (7)
C4	0.0253 (10)	0.0199 (10)	0.0199 (10)	−0.0008 (8)	0.0096 (8)	0.0008 (7)
C5	0.0253 (10)	0.0147 (10)	0.0216 (10)	−0.0018 (8)	0.0095 (8)	0.0016 (7)
C6	0.0270 (10)	0.0211 (10)	0.0217 (10)	−0.0021 (8)	0.0111 (8)	−0.0007 (8)

### Geometric parameters (Å, º)

**Table:**

Cl1—C1	1.764 (2)	Cl4—C4	1.769 (2)
Cl2—C1	1.777 (2)	Cl5—C4	1.772 (2)
Cl3—C1	1.764 (2)	Cl6—C4	1.773 (2)
O1—C2	1.398 (3)	O3—C5	1.396 (3)
O1—H1O	0.84 (3)	O3—H3O	0.76 (3)
O2—C3	1.236 (3)	O4—C6	1.235 (3)
N1—C3	1.332 (3)	N2—C6	1.332 (3)
N1—C2	1.440 (3)	N2—C5	1.439 (3)
N1—H1N	0.85 (2)	N2—H2N	0.78 (3)
C1—C2	1.550 (3)	C4—C5	1.549 (3)
C2—H2	0.96 (2)	C5—H5	0.99 (2)
C3—H3	0.99 (2)	C6—H6	1.00 (2)
			
C2—O1—H1O	112 (2)	C5—O3—H3O	110 (2)
C3—N1—C2	121.88 (17)	C6—N2—C5	122.84 (17)
C3—N1—H1N	116.3 (16)	C6—N2—H2N	118.0 (18)
C2—N1—H1N	120.8 (16)	C5—N2—H2N	118.7 (18)
C2—C1—Cl3	110.37 (14)	C5—C4—Cl4	110.64 (14)
C2—C1—Cl1	110.49 (14)	C5—C4—Cl5	109.84 (14)
Cl3—C1—Cl1	109.52 (11)	Cl4—C4—Cl5	109.91 (11)
C2—C1—Cl2	108.24 (14)	C5—C4—Cl6	108.82 (14)
Cl3—C1—Cl2	109.15 (11)	Cl4—C4—Cl6	108.79 (11)
Cl1—C1—Cl2	109.04 (11)	Cl5—C4—Cl6	108.80 (11)
O1—C2—N1	109.04 (17)	O3—C5—N2	109.32 (16)
O1—C2—C1	110.74 (16)	O3—C5—C4	111.15 (16)
N1—C2—C1	109.73 (16)	N2—C5—C4	109.14 (16)
O1—C2—H2	112.1 (13)	O3—C5—H5	111.4 (12)
N1—C2—H2	109.8 (14)	N2—C5—H5	109.6 (12)
C1—C2—H2	105.3 (14)	C4—C5—H5	106.2 (12)
O2—C3—N1	125.03 (19)	O4—C6—N2	125.30 (19)
O2—C3—H3	119.1 (14)	O4—C6—H6	120.9 (14)
N1—C3—H3	115.9 (14)	N2—C6—H6	113.7 (14)
			
C3—N1—C2—O1	−91.5 (2)	C6—N2—C5—O3	−95.7 (2)
C3—N1—C2—C1	147.08 (19)	C6—N2—C5—C4	142.52 (19)
Cl3—C1—C2—O1	−177.16 (14)	Cl4—C4—C5—O3	−58.44 (19)
Cl1—C1—C2—O1	−55.9 (2)	Cl5—C4—C5—O3	−179.96 (13)
Cl2—C1—C2—O1	63.45 (19)	Cl6—C4—C5—O3	61.03 (19)
Cl3—C1—C2—N1	−56.7 (2)	Cl4—C4—C5—N2	62.20 (19)
Cl1—C1—C2—N1	64.54 (19)	Cl5—C4—C5—N2	−59.31 (19)
Cl2—C1—C2—N1	−176.13 (14)	Cl6—C4—C5—N2	−178.33 (14)
C2—N1—C3—O2	−1.8 (3)	C5—N2—C6—O4	−2.1 (3)

### Hydrogen-bond geometry (Å, º)

**Table:**

*D*—H···*A*	*D*—H	H···*A*	*D*···*A*	*D*—H···*A*
O1—H1*O*···O2^i^	0.84 (3)	1.90 (4)	2.731 (2)	169 (3)
N1—H1*N*···O2^ii^	0.85 (2)	2.08 (3)	2.893 (2)	159 (2)
O3—H3*O*···O4^iii^	0.76 (3)	1.97 (3)	2.721 (2)	174 (3)
N2—H2*N*···O4^iv^	0.78 (3)	2.17 (3)	2.917 (2)	158 (2)
C6—H6···Cl4^v^	1.00 (2)	2.91 (2)	3.586 (2)	125 (2)

Symmetry codes: (i) −*x*, −*y*+1, −*z*; (ii) −*x*, *y*−1/2, −*z*+1/2; (iii) −*x*+1, −*y*, −*z*+1; (iv) −*x*+1, *y*+1/2, −*z*+1/2; (v) −*x*+1, *y*−1/2, −*z*+1/2.
